# A novel system to continuously estimate intradialytic blood pressure in real time

**DOI:** 10.1093/ndt/gfaf058

**Published:** 2025-03-24

**Authors:** Daniela Viramontes-Hörner, Paul Stewart, Jill Stewart, Maarten W Taal, Nicholas M Selby

**Affiliations:** Centre for Kidney Research and Innovation, Academic Unit for Translational Medical Sciences, School of Medicine, University of Nottingham, Derby, UK; Department of Renal Medicine, University Hospitals of Derby and Burton NHS Foundation Trust, Derby, UK; College of Science and Engineering, University of Derby, Derby, UK; Institute of Engineering, Computing and Advanced Manufacturing, University of Cumbria, Carlisle, UK; Centre for Kidney Research and Innovation, Academic Unit for Translational Medical Sciences, School of Medicine, University of Nottingham, Derby, UK; Department of Renal Medicine, University Hospitals of Derby and Burton NHS Foundation Trust, Derby, UK; Centre for Kidney Research and Innovation, Academic Unit for Translational Medical Sciences, School of Medicine, University of Nottingham, Derby, UK; Department of Renal Medicine, University Hospitals of Derby and Burton NHS Foundation Trust, Derby, UK

**Keywords:** arterial pulse wave, continuous blood pressure, haemodialysis, intradialytic hypotension, systolic blood pressure

## Abstract

**Background:**

Intradialytic hypotension (IDH) is a common complication of haemodialysis that is associated with adverse patient outcomes. We have developed a new non-invasive approach to continuously estimate systolic blood pressure (SBP) in real time during haemodialysis using pressure wave sensors in the extracorporeal circuit. We sought to compare the performance of our continuous real-time SBP estimator against brachial cuff SBP measurements.

**Methods:**

Single-centre, observational study conducted in 21 participants receiving haemodialysis with a functioning arteriovenous fistula, studied throughout two 4-h haemodialysis sessions. Time-averaged real-time SBP estimator values from the 5-s period immediately prior to each cuff measurement were compared with matched brachial cuff SBP values.

**Results:**

Mean age was 71 ± 11 years and median dialysis vintage was 20.0 months (interquartile range 12.5–63.5). Across 522 SBP comparison data points, mean brachial cuff SBP and real-time SBP estimate were 121.8 ± 27.1 mmHg and 123.7 ± 27.9 mmHg, respectively. Brachial cuff SBP and real-time SBP estimate were significantly associated (*r* = 0.825; *P* < .001). There was a low absolute mean difference between the brachial cuff SBP and the real-time SBP estimate of –1.9 ± 16 mmHg, and no evidence of systematic bias between measurements. Across all comparison points, 95% of estimator values were within 30% of the matched brachial cuff value, and 66% within 10% of the cuff value.

**Conclusions:**

A blood pressure estimator that runs in real time during haemodialysis using pressure wave sensors in the extracorporeal circuit and avoiding additional sensor-burden on patients has good performance in tracking intradialytic SBP when compared against brachial cuff measurements, supporting its further development and larger scale testing.

## Graphical Abstract

**Graphical Abstract:**
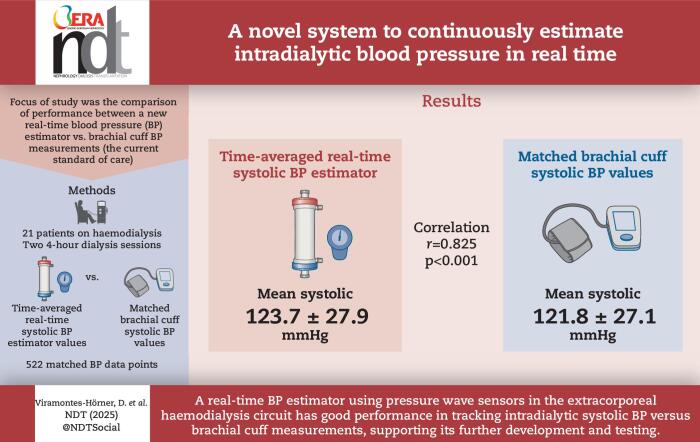

KEY LEARNING POINTS **What was known:**

- We have developed a new method to continuously estimate systolic blood pressure (SBP) during haemodialysis (when an arterio-venous fistula is used).

**This study adds:**

- In a clinical study, we have shown good agreement between real-time SBP estimator and brachial cuff SBP measurements.

**Potential impact:**

- Further development of the technology and larger scale clinical testing of this approach to continuously estimate intradialytic SBP is now warranted.

## INTRODUCTION

Haemodialysis is often complicated by intradialytic hypotension (IDH), with 10%–40% of treatments affected [1, 2]. IDH causes unpleasant symptoms for patients and can lead to inadequate dialysis due to shortened treatments or failure to achieve ultrafiltration goals. Furthermore, IDH is strongly associated with increased mortality, whether defined as the magnitude of fall in blood pressure (BP) [3], or the nadir BP value during dialysis [4]. Mechanistically these associations can be explained, at least in part, by reduced perfusion in vulnerable vascular beds during dialysis. Specifically, repeated episodes of myocardial and cerebral ischaemia associated with IDH have been shown to lead to persistent organ dysfunction and are independently associated with increased mortality [5].

Current clinical practice involves measuring BP every 30–60 min during dialysis using brachial oscillometric cuff measurements [6]. However, this approach has a number of limitations. Periodic BP measurement means that management of IDH is largely reactive, instituted only after BP has already fallen and with considerable delays before interventions are implemented. It is also widely recognized that BP measurement in busy dialysis units often falls short of ideal, with measurements taken less often than recommended or performed poorly (e.g. cuffs placed over clothing resulting in inaccurate readings) [7]. Continuous BP measurement during dialysis would allow earlier detection and intervention, and may also facilitate new approaches for real-time prediction of IDH [8, 9]. Until now, this has relied on techniques such as digital artery photoplethysmography, which is restrictive for patients, is not suitable outside of research settings and may not function well in a significant proportion of patients.

We have developed a new approach to continuously estimate systolic blood pressure (SBP) in real time during dialysis using additional pressure wave sensors in the extracorporeal circuit, without additional sensors attached to the patient. We have previously reported its methodology and initial proof of concept [10], as well as an update to better model variance in physiological parameters over time [11]. Here, we report a prospective study in which we sought to compare performance of our continuous real-time SBP estimator against brachial cuff SBP measurements, the current standard of care.

## MATERIALS AND METHODS

### Study design and population

We performed a single-centre, observational study conducted between February and November 2023 in the Department of Renal Medicine, Royal Derby Hospital. People with kidney failure receiving regular haemodialysis who were ≥18 years of age, dialyzed at least three times per week for 4 h and had a functioning arteriovenous fistula (AVF) were eligible. Exclusion criteria included arteriovenous grafts and poorly functioning AVFs [i.e. clinical problems such as high arterial/venous pressures, or two or more consecutive Qa (AVF blood flow) values <500 mL/min as estimated using ionic dialysance [12]]. Participants were studied throughout two haemodialysis treatments, with the aim of comparing intradialytic SBP values from a newly developed real-time continuous BP estimator against SBP values measured by a standard arm cuff (current standard of care).

This study was conducted according to the guidelines laid down in the Declaration of Helsinki and all procedures involving patients were approved by the local Research Ethics Committee (West Midlands Coventry and Warwickshire, REC reference: 17/WM/0080). Written informed consent was obtained from all participants.

Baseline demographic characteristics including age, sex and ethnicity, as well as dialysis vintage (i.e. time since first dialysis treatment), routine blood results, medical history, use of anti-hypertensive medication, post-dialysis weight, body mass index and dialysis parameters including type of dialyser and vascular access, AVF blood flow (Qa) values and needle gauge were collected from electronic medical records.

### Continuous blood pressure measurement

**Figure 1:**
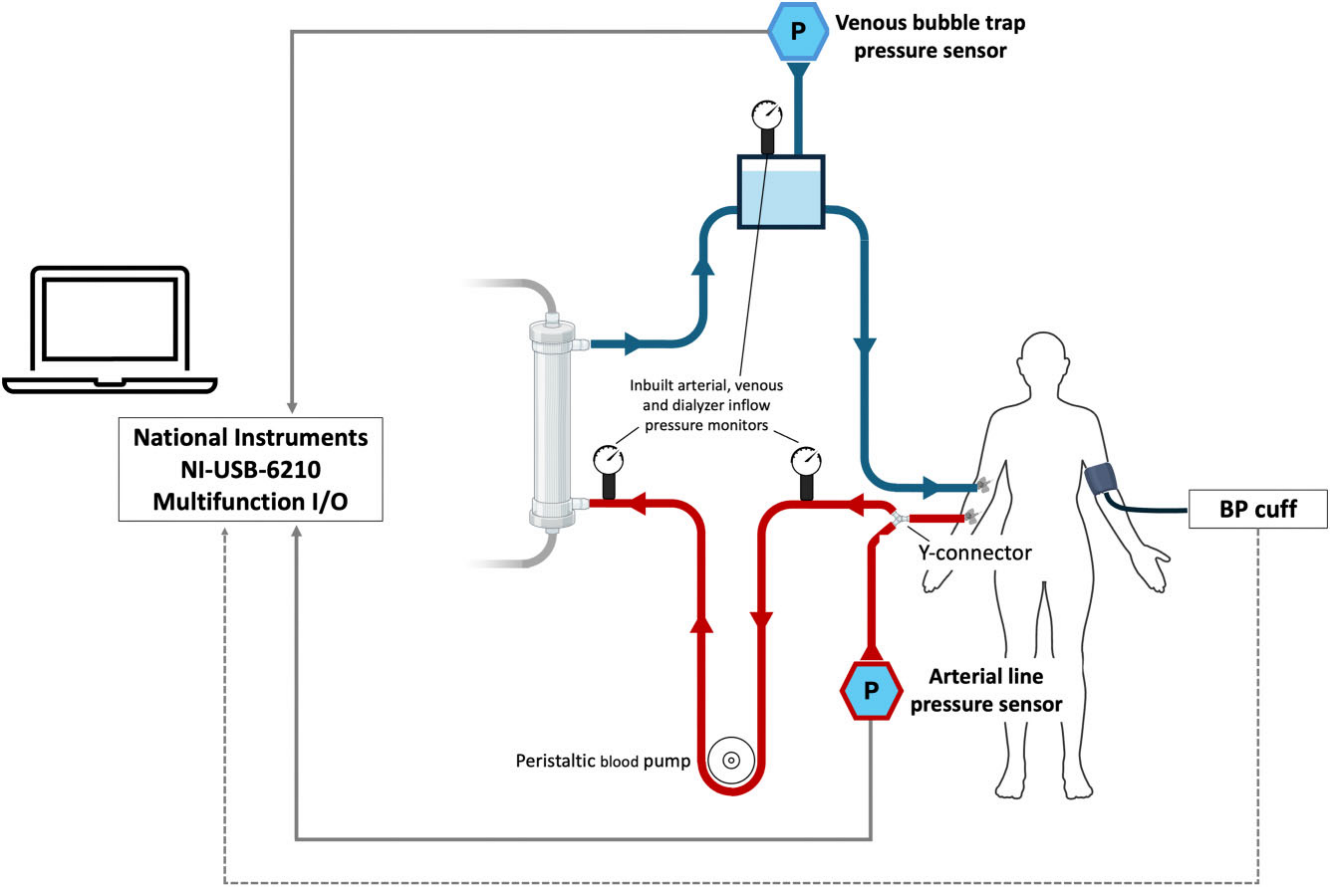
Diagram illustrating the placing of additional pressure sensors in the dialysis circuit to enable real-time continuous SBP estimation.

For each study session, participants had continuous monitoring of SBP throughout a complete haemodialysis treatment via the real-time BP estimator, as previously described [11]. Briefly, pressure sensors were attached to the extracorporeal circuit, one to a y-connector close to the arterial needle to derive a continuous arterial pressure waveform, and a second connected to a port on the venous bubble trap to record the pressure waveform generated by the peristaltic blood pump (Fig. 1). The pressure sensors were separated from participants’ blood by a 0.2-µm sterile filter and a non-sterile sealed membrane. Continuous pressure data were stored and integrated in an attached computer using a Simulink model in Matlab software. The Simulink model applies an iterative learning run-to-run modelling methodology originally developed for process control engineering applications to generate a parameterized BP model to continuously estimate SBP in real time, incorporating real-time data from pressure sensors, as well as intermittent data from the most recent brachial BP cuff measurement (taken every ∼20 mins) [11].

### Primary analysis

To determine if the real-time BP estimator was able to track changes in intradialytic SBP, we compared the time-averaged value generated by the estimator from the 5-s period immediately prior to each SBP cuff measurement with the corresponding SBP cuff value across all measurement pairs from both study sessions for each participant.

### Statistical analyses

SPSS version 28.0 (IBM Corporation, Chicago, IL, USA) was used for data management and to perform all statistical analyses. Data are presented as mean ± standard deviation, median [interquartile range (IQR)] or percentages, as appropriate. A *P*-value <.05 was considered to have statistical significance. Pearson's correlation coefficient was used to determine the significance and strength of the association between brachial cuff SBP and the real-time SBP estimate (time-averaged value from the 5-s period immediately prior to SBP cuff measurement). Bland–Altman analysis was used to compare the agreement between the brachial cuff SBP and the real-time SBP estimate and assess for bias [13]. Due to multiple measures for each individual, we performed a linear mixed-effects model in which the mean value of both measurements was used as a fixed effect and the variability between subjects was estimated as random effects, and used the outputs of this model to re-estimate bias, upper and lower limits of agreement [14]. The intraclass correlation coefficient was also calculated. The P10 and P30 values, defined as the percentage of real-time SBP estimator values within 10% and 30% of brachial cuff SBP values, respectively, were also calculated. We defined IDH as brachial cuff SBP of ≤100 mmHg. For all IDH episodes, we calculated the proportion of matched SBP estimator values that were ≤100 mmHg and ≤110 mmHg and calculated positive predictive value, negative predictive value, sensitivity and specificity at each threshold.

## RESULTS

### Participant characteristics

A total of 21 participants were included in the study, generating 42 completed monitored haemodialysis sessions and 522 BP comparison data points (brachial cuff SBP and a matched SBP estimator value from the 5-s period immediately prior to SBP cuff measurement). The median number of paired SBP readings per patient was 24 (IQR 22–28).

Demographic, clinical and biochemical participant characteristics are shown in Table 1. Mean age was 71 ± 11 years, and median dialysis vintage was 20.0 months (IQR 12.5–63.5). Most of the participants were male (57%) and of White ethnicity (76%). Prevalence of diabetes and cardiovascular disease were 33% and 52%, respectively. There was an even split between participants with brachiocephalic (10) and radiocephalic (11) AVF. AVF function was good with a median AVF blood flow (Qa) of 598 mL/min (IQR 390–1096). Fifteen (71%) participants used 14-gauge needles and the remainder used 15-gauge needles.

**Table 1:** Participant characteristics.

Variable	(*n =* 21)
Age (years)	71 ± 11
Male [*n* (%)]	12 (57)
White ethnicity [*n* (%)]	16 (76)
Diabetes [*n* (%)]	7 (33)
Cardiovascular disease [*n* (%)]	11 (52)
Previous kidney transplant [*n* (%)]	1 (5)
Dialysis vintage (months)	20.0 (12.5–63.5)
Antihypertensive medication [*n* (%)]	
Angiotensin converting enzyme inhibitors	3 (14)
Calcium channel blockers	3 (14)
Beta blockers	7 (33)
Fistula blood flow assessment (Qa)	598 (390–1096)
Dialyzer [*n* (%)]	
Medium cut-off	19 (90.5)
High-flux	2 (9.5)
Vascular access type [*n* (%)]	
Brachiocephalic arteriovenous fistula	10 (48)
Radiocephalic arteriovenous fistula	11 (52)
Needle gauge [*n* (%)]	
2 × 14 g	15 (71)
2 × 15 g	6 (29)
Serum albumin (g/dL)	2.9 ± 0.4
Haemoglobin (g/dL)	11.7 ± 0.1
Post-dialysis weight (kg)	75.0 ± 16.8
Body mass index (kg/m^2^)	26.8 ± 4.5

Data are expressed as mean ± standard deviation, median (interquartile range) or numbers (percentages) as appropriate. Presence of cardiovascular disease was defined by at least one of the following events/diagnoses at the baseline assessment: myocardial infarction, cerebrovascular disease/stroke, peripheral vascular disease, heart failure, coronary artery disease/surgery and ischaemic heart disease.

### Correlation, agreement and accuracy of blood pressure measurements

**Figure 2:**
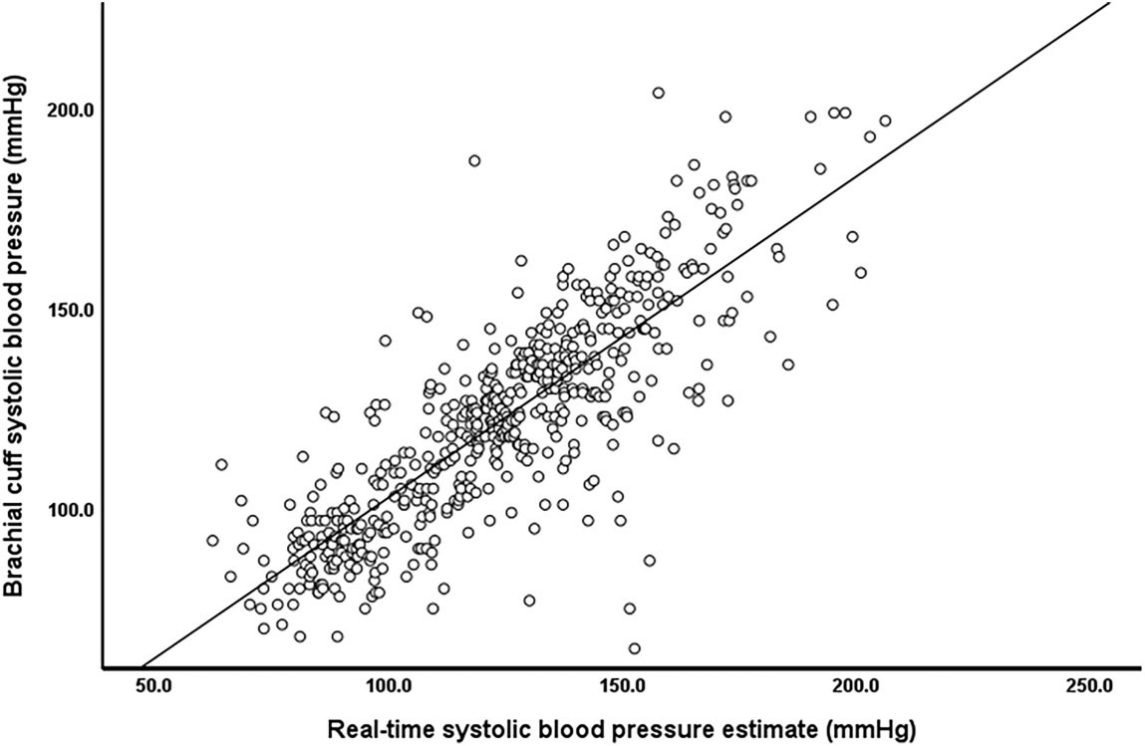
Pearson's correlation between brachial cuff SBP measurement and real-time SBP estimate.

Across all measurements, mean brachial cuff SBP and real-time SBP estimate were 121.8 ± 27.1 mmHg and 123.7 ± 27.9 mmHg, respectively. There was a strong and significant association between the brachial cuff SBP measurement and the real-time SBP estimate (*r =* 0.825; *P* < .001, Fig. 2).

**Figure 3:**
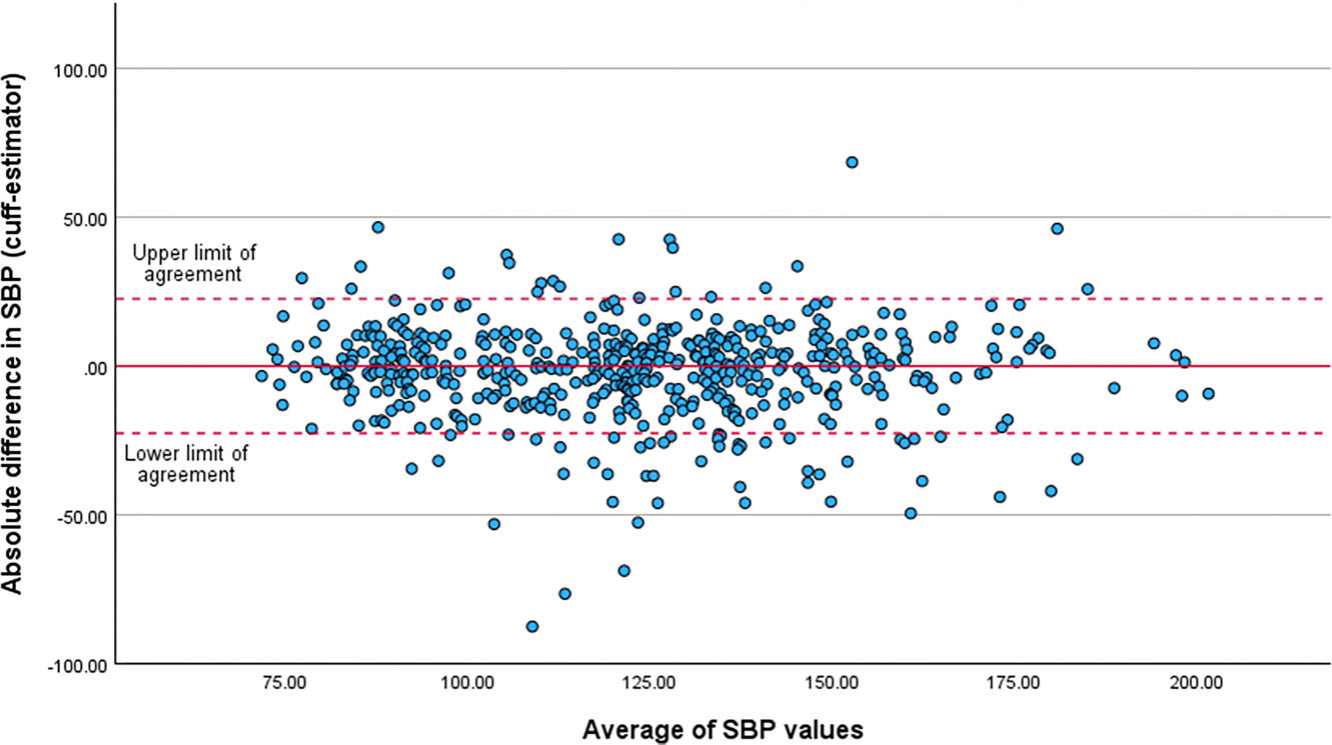
Bland and Altman plot comparing SBP measured by brachial cuff and real-time estimator. The middle solid line represents the mean absolute difference between measurements (–1.9 ± 16 mmHg). The dashed lines represent the upper and lower limits of agreement which were derived from the linear mixed-effects model (±22.6 mmHg). As reference, the values of the upper and lower limits of agreement based on ±1.96 standard deviation of differences between individuals was ±23.0 mmHg, not taking account of multiple measures per individual.

Figure 3 shows the Bland–Altman plot of the difference between the two SBP measures at each data comparison point (brachial cuff SBP – real time SBP estimator) against the average of the two. There was a low absolute mean difference between the brachial cuff SBP measurement and the real-time SBP estimate of –1.9 ± 16 mmHg, and no evidence of systematic bias between measurements. There was some degree of dispersion of the data, and using absolute numbers (sign removed), the mean difference between the two SBP measures was 11.5 ± 11 mmHg. The intraclass correlation coefficient between the two methods was 0.9 [95% confidence interval (CI) 0.88–0.92]. The P30 and P10 data demonstrated good agreement between the two SBP measures, with 95% (95% CI 93%–97%) of estimator values within 30% of the matched brachial cuff value and 66% (95% CI 62%–70%) within 10% of the cuff value.

A total of 131 (25%) of the brachial cuff SBP measurements were ≤100 mmHg and categorized as IDH. Using a threshold of ≤100 mmHg, the positive predictive value of the estimator was 79.1% (95% CI 71%–85%), negative predictive value 94.1% (95% CI 91%–96%), sensitivity 82.2% (95% CI 75%–87%) and specificity 92.9% (90%–95%). At a threshold of ≤110 mmHg, positive predictive value was higher at 90.3% (95% CI 84%–94%), negative predictive value was lower at 86.6% (95% CI 83%–90%), with sensitivity of 70.0% (95% CI 63%–76%) and specificity of 96% (95% CI 94%–98%).

## DISCUSSION

IDH remains a common and serious consequence of haemodialysis. Intermittent brachial cuff BP measurements during dialysis have significant limitations and clinical practice has not progressed for several decades. We have developed a new technology to continuously estimate SBP in real time using additional pressure sensors in the extracorporeal circuit but without additional sensors applied to the patient, and here have demonstrated that it performs well when compared with brachial cuff measurements.

Changes in response to ultrafiltration and reducing plasma osmolarity during dialysis manifest as IDH when the resulting fall in circulating volume overwhelms compensatory haemodynamic mechanisms. IDH is easily detected when accompanied by symptoms, but often is asymptomatic and may not always be recognized. This risk of under-detection is important when considering the strong associations between IDH and adverse patient outcomes [3, 4]. Even very short-term variations in BP may be undesirable, having been shown to associate with biomarkers of cardiac injury and subclinical ischaemic change on brain imaging [15]. Developing technology suitable for clinical environments to continuously measure BP during dialysis would therefore be a significant advance from the current status quo, allowing earlier detection and intervention for IDH episodes. Other advantages may also be realized if continuous BP data allows modelling for real-time prediction of IDH to facilitate pre-emptive intervention, or facilitates new approaches to better characterize intradialytic BP variation.

Previous attempts to measure BP continuously during dialysis have included a wearable photoplethysmography wrist band [16], an electronic stethoscope attached to the AVF [17], bioimpedance cardiography [18] or, most commonly, a finger cuff attached to the non-AVF hand to measure the pulse wave in the digital artery [9, 19–24]. None of these has translated to clinical application, likely because of inaccuracy of measurement or the requirement for significant additional monitoring attached to the patient. Additional sensors applied directly to patients have disadvantages and may not be acceptable, for example if they are uncomfortable or if they restrict use of the non-AVF arm. Problems may also arise if patient movement interferes with data acquisition, or if technology fails in the face of common scenarios such as vascular calcification and increased vascular stiffness. Our approach, which does not involve additional sensors applied to the patient, avoids most of these issues. The pressure sensor in close proximity to the AVF allows for the derivation of an arterial pressure waveform. The pressure sensor connected to the venous bubble trap helps to distinguish this from the dominant pressure waveform arising from the peristaltic blood pump, as well as providing a way for the system to incorporate changes in the blood pump speed during dialysis. Our system is able to display the BP estimator value in real time, which is essential for use in clinical settings, and incorporating the most recent cuff value during dialysis allows the system to adapt to unmeasured time-varying physiological parameters.

Whilst the gold standard comparator would be BP derived from an intra-arterial catheter, this is not feasible in dialysis populations. In the absence of this, we therefore compared our non-invasive continuous BP estimator against brachial cuff measurements taken approximately every 20 min throughout dialysis. Our results showed the real-time estimator performed well with readings that were well correlated with brachial cuff measures, with a small mean difference between the two and without systematic bias. Nevertheless, the limits of agreement between the two measures were relatively wide. Without a gold-standard comparator, disagreements may arise from measurement variation in either technique. It is possible that some of the variation between the two methods may arise from inaccurate brachial cuff readings, but conversely would also arise if the estimator has not correctly tracked true BP. The clinical implications of this are most important in terms of ensuring that true IDH episodes do not go undetected, and it was therefore notable the estimator was able to detect the majority of IDH episodes, particularly at the higher SBP threshold (positive predictive value >90). Accuracy of the estimator would also be important in terms of using the data for predictive modelling and in avoiding alert fatigue that may arise from false positive alerts. The proportion of BP measurements that were categorized as IDH was relatively high at 25%, likely in part because more frequent measurement of BP is likely to result in greater detection of hypotensive episodes, the majority of which are asymptomatic. This has been shown in other studies using continuous BP measurement, with one study reporting IDH in 37.5% of patients [18].

Our results should be interpreted in the light of some weaknesses. Participants were selected to have a well-functioning AVF, and it is uncertain how significant flow-restricting stenoses would have affected the performance of the real-time BP estimator. This will be subject of future work. As discussed, we have not been able to compare the real-time estimator against BP values from an intra-arterial catheter, as the inconvenience and risks of doing so in dialysis patients are hard to justify. In addition, our current prototype system requires wired connections between the pressure sensors and computer, so only one participant can be studied at a time. Next stage prototypes are in development that will have wireless connections so that a central computer can be connected to pressure sensors across multiple dialysis machines. Finally, our definition of IDH was based solely on BP threshold and did not take account of patients’ symptoms, and further studies are required to more robustly study the detection of IDH, along with studies to evaluate the clinical impact of continuous intradialytic BP estimation, for example whether this approach will result in earlier and more effective interventions to correct or prevent IDH.

In conclusion, we have developed a BP estimator that runs in real time during dialysis using additional pressure sensors in the extracorporeal circuit, and avoiding additional sensor burden on patients. When compared against regular brachial BP measurements, it has good performance in tracking intradialytic SBP and this supports its further development and larger scale testing. Planned next steps are to develop a computer algorithm to predict IDH in real time and to assess the impact of using this approach to enable pre-emptive interventions to prevent IDH.

## Data Availability

Data sharing requests will be considered on application to investigators
